# The Boost study: design of a school- and community-based randomised trial to promote fruit and vegetable consumption among teenagers

**DOI:** 10.1186/1471-2458-12-191

**Published:** 2012-03-14

**Authors:** Rikke Krølner, Thea Suldrup Jørgensen, Anne Kristine Aarestrup, Anne Hjøllund Christiansen, Anne Maj Christensen, Pernille Due

**Affiliations:** 1 Centre for Intervention Research in Health Promotion and Disease Prevention, University of Southern Denmark, National Institute of Public Health, Øster Farimagsgade 5A 2nd floor, 1353 Copenhagen K, Denmark; 2 The Danish Institute for Health Services Research (DSI), Dampfærgevej 27-29, 2100 Copenhagen Ø, Denmark

**Keywords:** fruit, vegetables, school, intervention, adolescents, community, availability, accessibility, Intervention Mapping

## Abstract

**Background:**

The aim of the Boost study was to produce a persistent increase in fruit and vegetable consumption among 13-year-olds. This paper describes the development, implementation and evaluation of a school-and community-based, multi-component intervention guided by theory, evidence, and best practice.

**Methods/design:**

We used the Intervention Mapping protocol to guide the development of the intervention. Programme activities combined environmental and educational strategies and focused on increasing access to fruit and vegetables in three settings: School: Daily provision of free fruit and vegetables; a pleasant eating environment; classroom curricular activities; individually computer tailored messages; one-day-workshop for teachers. Families: school meeting; guided child-parent activities; newsletters. Local community: guided visits in grocery stores and local area as part of classroom curriculum; information sheets to sports-and youth clubs.

The Boost study employed a cluster-randomised controlled study design and applied simple two-stage cluster sampling: A random sample of 10 municipalities followed by a random sample of 4 schools within each municipality (N = 40 schools). Schools were randomised into a total of 20 intervention-and 20 control schools. We included all year 7 pupils except those from school classes with special needs. Timeline: Baseline survey: August 2010. Delivery of intervention: September 2010-May 2011. First follow-up survey: May/June 2011. Second follow-up survey: May/June 2012. Primary outcome measures: Daily mean intake of fruit and vegetables and habitual fruit and vegetable intake measured by validated 24-hour recall-and food frequency questionnaires. Secondary outcome measures: determinants of fruit and vegetable intake, positive side-effects and unintended adverse effects. Implementation was monitored by thorough process evaluation.

**Discussion:**

The baseline data file included 2,156 adolescents (95%). There was baseline equivalence between intervention-and control groups for sociodemographics, primary outcomes, and availability at home, school and sports-and youth clubs. Significantly larger proportions of pupils in the control group had parents born in Denmark. The study will provide insights into effective strategies to increase fruit and vegetable intake among teenagers. The study will gain knowledge on implementation processes, intervention effects in population subgroups with low intake, and opportunities for including local communities in interventions.

**Trial registration:**

Current Controlled Trials ISRCTN11666034.

## Background

Many adolescents in western countries, including Denmark, do not meet the World Health Organisation's (WHO) recommendations of a daily intake of at least 400 grams of fruit and vegetables (FV) [1,2]. Especially the vegetable intake lags behind [1,2]. Moreover, FV intake among children decreases as they enter the teen years [3]. The international Health Behaviour in School-Aged Children (HBSC) surveys among 11-, 13-and 15-year-olds show a significant decline in proportions of children eating FV daily by age in almost all countries and most consistently among boys [4,5]. The reason for the decline from age 11 to 15 is unknown. Well-established modifiable determinants among children and adolescents are home availability, parental intake and children's FV taste preferences [3] but these factors may not be sufficient to explain age differences in FV intake. Qualitative studies suggest that peers, short term outcome expectancies, situational norms and different aspects of availability such as variety, convenience and attractiveness of FV and access to unhealthy food may be important for teenagers' FV intake [6]. Furthermore, the transition to secondary school may involve new habits and influences for adolescents as they sometimes change school environments, and may exercise more autonomy over their own food choice. They have increased opportunities for eating unhealthily as they are often allowed to leave school during breaks, spend more time and meals together with their friends and have their own money to spend which limits their parents' influence as role models and caretaker for their dietary behaviour. Teenagers' food choice may function as a statement of independence and detachment from parents as well as a symbolic marker of social identity and belongingness to certain peer groups [6-9]. FV intake in the early teens may be crucial as longitudinal studies show that the frequency of FV intake at the age of 13-14 years is a good indicator of frequency of consumption in young adulthood [10,11].

Few interventions have targeted secondary school-aged children (13-18 years of age) and with mixed results [12,13]. Reviews of intervention studies among children and adolescents have concluded that theory-based, multi-component school-based approaches of a long duration and intensity combining educational and environmental strategies in school with parent involvement are most effective in increasing FV consumption [12,14,15].

### Availability of FV

The importance of availability of FV for adolescents' FV intake has been confirmed in literature reviews of both qualitative studies, observational studies and intervention studies [3,6,12,14,15]. Intervention studies in Norway, Belgium, and Canada have shown that free provision of fruit and/or vegetables in schools can increase adolescents' fruit and/or vegetable intake [16-21]. Studies from Norway suggest that free FV programmes are more effective than subscription programmes in increasing fruit consumption [18]. Subscription programmes may increase social inequality in FV intake as subscribers include mainly high income families whereas free provision increases intake among adolescents from families of low socioeconomic position (SEP) as well [18]. Most interventions have reported larger effects for fruit intake than vegetable intake [21-24] and it remains a challenge to develop effective methods to increase vegetable consumption.

Many intervention studies have only succeeded in increasing FV intake in school, not the total daily intake which may be explained by minimal parental involvement and by the fact that FV provided in schools replace some of the FV eaten at other times of the day [19,22]. Implementation of home components is challenging as few parents tend to participate [25,26]. Home availability and accessibility, parental intake, and SEP are all consistently associated with adolescents' FV consumption [3,27]. It is therefore important to develop strategies which increase parental involvement and support to adolescents' FV intake [28]. The Pro Children multi-component intervention programme which included a family component (parental newsletters, family home assignments) together with FV provision (free or subscription) and a classroom curriculum had a significantly positive effect on total daily fruit-, vegetable-and FV intake among 10-11-year-olds in Norway, the Netherlands and Spain at 8-month follow-up [21].

Multi-component interventions have been suggested to be more effective than single component strategies due to synergistic effects between educational and environmental strategies [29]. Other studies suggest that a single strategy which only increases access to FV is equally or even more effective in increasing adolescents' FV intake [17,30]. Intervention components such as curricular activities are not always implemented as intended. Low fidelity and small doses delivered challenge the validity of assessments of the effectiveness of multi-component interventions [31-33]. Therefore, thorough process evaluation, including detailed monitoring of the implementation is important [32,33].

Computerised individualised tailoring of nutrition education seems to be an effective method of nutrition interventions among adults [34,35]. The evidence for the effectiveness among adolescents is unclear [36-38]. The method has the potential to be an effective intervention strategy as it is a popular intervention activity in this age group [39]. A review from 2010 concluded that dynamically tailored interventions (ipsative feedback) had increased efficacy over time as compared with tailored interventions based on one assessment only [40]. However, this approach has not been widely tested among adolescents.

Many intervention studies suffer from methodological limitations such as lack of randomisation, lack of baseline measurements, low response rates, inadequate adjustment for clustering of schools, short duration of intervention, short follow-up period, lack of explicit theoretical basis, lack of an explicit programme theory (causal model), and lack of process evaluation [13,36,38,41]. Furthermore, evaluation studies of multi-component interventions often report the collective, synergistic effect of the intervention as it is challenging to disentangle the relative importance of specific components. Evaluation of different combinations of strategies using multi-arm controlled designs requires substantial statistical power [19,20] and there is a need for new approaches to decompose the effects.

Many FV intervention studies have used determinants as proximal outcomes and measured changes in these. Fewer studies have examined if changes in FV intake were preceded by changes in determinants (mediation analyses) and even fewer have linked changes in determinants to implementation of specific intervention components. In order to make valid interpretations of the proximal outcomes (determinants) of the intervention and examine which methods are efficient in changing each of these determinants we need to know whether the specific components were effectively administered [32,42]. Moreover, there is a lack of insight in programme reach and differential effects on proximal (determinants) and distal outcomes (FV intake) in population subgroups with low intake such as boys and low income groups. Few studies have examined how and if these subgroups benefit from interventions [43] and if intervention components reach all subgroups equally (effect modification analyses).

It is important to promote access to FV in the multiple settings of adolescents' lives to consistently support them in making healthy food choices throughout the day. Despite this fact most nutrition interventions have addressed the school-or home environment and not the neighbourhood and leisure time activities. There is a great potential in sports-and youth clubs to promote healthy eating habits to adolescents in their leisure time [44]. Cross-sectional studies from Australia, Finland and Denmark have pointed at factors promoting unhealthy eating habits in sports clubs [44-47]. The same challenges are found in youth clubs in Denmark [48]. A Cochrane review from 2008 concluded that there is a lack of controlled studies evaluating policy interventions aiming at enhancing health behaviours through sporting organisations [49].

Given the rationale above, the aim of the Boost study was to develop, implement and evaluate a comprehensive school-based and community-integrated intervention which produces a persistent increase in FV consumption among teenagers. The key component of the intervention programme was to improve the determinants for FV intake, especially to enhance availability of FV in the multiple settings in which teenagers take part. The developmental work was to be guided by theory, evidence and best practise. This paper presents the development of the intervention, the design of the effectiveness study, and the baseline characteristics of pupils at intervention-and control schools.

### Conceptualisation, design and methods

The Boost intervention builds on a socio-ecological framework which recognises adolescents' eating behaviour such as intake of FV as affecting and being affected by multiple levels of interacting influences [50]. Ecological models emphasise the structural, physical and political context while incorporating social and psychological influences. A practical implication of the ecological framework is multilevel interventions which use multiple strategies to increase adolescents' consumption of FV and ensure that healthy messages are consistent and coherent throughout the settings in which the adolescents take part. The Boost study recognises the importance of availability of FV as an enabling factor. We used main principles from the Intervention Mapping protocol to plan the intervention, implementation and evaluation in a systematic fashion, and the study was guided by advice from a steering committee of internationally recognised experts from the field [51]. The six consecutive steps of the Intervention Mapping planning process are described below. In reality, we did not use the protocol rigidly but moved back and forth between the steps as suggested by the authors (iterative planning process) [51].

### Needs assessment (step 1)

As part of the needs assessment a national planning group was established, the programme theory was drawn up, target group and programme objectives were specified and the needs, values and views of the target group were analysed.

#### The planning group

The planning group consisted of parents of teenagers, practitioners and experts with experiences in implementing school fruit breaks, public food provision, participatory research with teenagers, teaching, development of teaching material, and lobbying with stakeholders. The planning group discussed the scope and feasibility of initial ideas of the intervention programme, assisted the project group in making contacts with important stakeholders and was included in deciding the final programme theory and intervention model.

#### Target groups

We narrowed our target group to 13-year-olds (school year 7) because of the decline in FV intake observed in this age group [4,5]. Moreover most intervention studies had previously focused on younger children [12,13]. The Boost intervention was designed to reach all 13-year-olds independent of sociodemographic characteristics (population-based preventive strategy) but the special needs of vulnerable groups were taken into account by target group analysis among boys and adolescents from low income homes.

#### Target group analysis

We conducted two gender-homogeneous focus group discussions, one with boys (N = 8) and one with girls (N = 8) from year 7 in a Danish school with a high proportion of children from low income families. The focus groups were designed to explore perceptions and experiences with barriers and facilitators for eating FV, as well as the target groups' views on the relevance and appeal of our initial intervention ideas and strategies. In agreement with studies from other countries [6], the adolescents emphasised home availability, school availability, price, taste, food preparation, and appeal as influencing their intake. To help guide our selection of communication channels and development of visual identity and intervention tools two school classes of year 7 pupils from the same school completed a short questionnaire on their favourite dishes, hobbies, music, books, use of cell phone, time spent on computer, and memberships of internet forums and sports-and youth clubs. Parents at the same school were invited to participate in a parent focus group to illuminate parental barriers and ways to involve parents in interventions. Only one parent accepted our invitation and was interviewed face-to-face. Finally, we carried out observations at one school during lunch breaks to observe the year 7 pupils habits and activities.

### Setting

#### The Danish school system

All children are entitled to free tuition at Danish municipal primary and lower secondary school. This tuition includes a one-year, pre-school class followed by nine years of primary and lower secondary school. Private schools exist where a small part of the tuition is paid for by the parents. Most children in Denmark (86% in 2008) attend the municipal primary and lower secondary school [52]. Children automatically attend a municipal primary and lower secondary school in the area where the family lives. Children will often stay in the same school class throughout their school education. Schools have a board that includes school representatives and representatives elected by the parents themselves. Pupils have the opportunity to influence their school by forming pupil councils to inform important school decisions. There is no national provision of school meals. Children usually bring their own lunch bag or buy lunch in a school canteen if available [52,53].

#### Outside school hours

##### Youth schools, recreational junior-and youth clubs

All Danish municipal authorities have youth schools for adolescents aged 13 to 18. The Youth School is a special Danish education form, where youth can supplement their school education in their spare time. Enrolment is voluntary and tuition is free. Youth schools are open in the afternoon and evening, and here it is possible to take academic and creative subjects such as music, photography, learn about IT or simply hang out with other young people. Some municipal authorities also have recreational clubs adolescents can attend when they have outgrown municipal after-school centres or after-school care schemes some of which must be paid for by participants [53]. Here we use 'youth clubs' as an umbrella term for both youth schools and recreational clubs.

##### Local sports associations and sports clubs

In Denmark almost two thirds of all children and adolescents are engaged in organised sports in their leisure time. They are all run by a committee elected by the members. Association life is mostly based on voluntary, unpaid work by managers and coaches [54].

#### Project name and visual identity

The project was named 'Boost' to indicate energy and power. The intervention was designed to boost the adolescents' FV intake and energy level. Names in English appeal to Danish teenagers and the intention was to make the 13-year-olds perceive the project as 'cool'. The logo was also designed to express vitality and with a humorous tone to appeal to the adolescents.

#### Programme theory and outcomes

**Figure 1:**
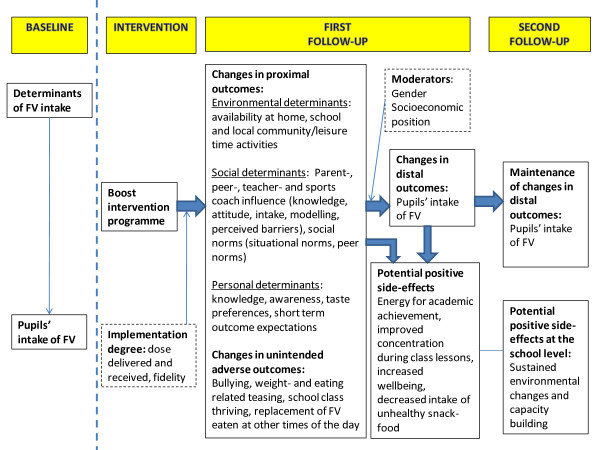
**Programme theory of the hypothesised causal relationship between the Boost intervention programme, proximal and distal outcomes and side-effects**.

The programme theory (Figure 1) was guided by systematic reviews of determinants of adolescents' FV intake, reviews of intervention studies, theory, experiences from recent successful or innovative intervention studies, especially the international Pro Children study and the Norwegian Health In Adolescents (HEIA) study [3,6,39,55-57], input from the planning group and international steering committee, and discussions with the target group. In agreement with best practice, the programme theory outlined the causal chain which explains how the intervention is expected to impact distal outcomes (FV intake) through changes in proximal outcomes (determinants). We conducted a brainstorm and prioritisation of important and changeable determinants of adolescents' FV consumption identified from systematic literature reviews [3,6]. As shown in Figure 1 the intervention was designed to produce changes in environmental, social and personal determinants. We hypothesised that the effect would differ by gender and SEP because of the low FV intake among boys and low income children [3]. Likewise we hypothesised that the effect of the intervention would differ by implementation degree.

Furthermore, the programme theory (Figure 1) mapped out unintended adverse effects as well as anticipated potential positive side effects of the intervention [58]

Assessment of adverse effects is often neglected in effectiveness studies [58]. It is relevant to explore whether intervening in the adolescents' life produces any unintended adverse effects as suggested in Figure 1. Eating behaviours may be a vulnerable issue among teenagers and we paid special attention to this in the development of project logo and curricular activities. We did not want to contribute to promotion of a certain body image or discrimination of certain groups e.g. adolescents with overweight problems or unhealthy eating habits. Also we did not want to exhibit low income families in curricular activities. For instance, home assignments on exploring the FV supply at home and monitoring parents' and siblings' FV intake for a week were not planned for class discussion, but for discussion within the family only.

### Matrices (step 2)

In step 2 we aimed at developing a comprehensive intervention reaching all important determinants specified in the programme theory (Figure 1). Matrices were created according to the Intervention Mapping protocol and change objectives were decided. Tables 1, 2 display a small section of two of the educational and environmental change objectives.

**Table 1:** Behavioural performance objectives: determinants, change objective, theoretical methods and practical applications

Behavioural performance objective:	Determinants:			
	
	Awareness	Knowledge	Attitude	Parental intake
Become aware of own FV intake in relation to national recommendations	*Change objective: *Assess/evaluate own FV intake	*Change objective: *Know the national FV recommendations Know the size of one portion of vegetables	*Change objective: *Change perceptions of the importance and relevance of eating FV	*Change objective: *Become aware of parents FV intake

	*Theoretical methods: *Feedback	*Theoretical methods: *Information Skills training	*Theoretical methods: *Reinforcement	*Theoretical methods: *Social comparisons

	*Practical application: *Register own FV intake as part of curricular activities (computer tailoring)	*Practical application: *Class-based educational activities or schoolchild-parent assignment: How much does a portion weigh? Gain knowledge of Recommendations Weigh FV	*Practical application: *Experience immediate positive outcomes of eating FV versus e.g. chocolate Class discussion about immediate benefits of eating FV	*Practical application: *Register parents' FV intake as part of homework

**Table 2:** Environmental performance objectives: determinants, change objective, theoretical methods and practical applications

Environmental performance objective:	Determinants:			
	
	Availability	Accessibility	Taste preferences	Peers/social norms
Create a more supporting school environment for eating FV	*Change objective: *Provide free FV daily	*Change objectives: *Allocate time to intake of FV Provide kitchen tools	*Change objective: *Challenge adolescents' taste preferences by exposing them to a variety of FV	*Change objective: *Create a pleasant eating environment Change adolescents' perception of appropriate time, settings and situations for eating FV Create role models for eating FV among classmates

	*Theoretical methods: *Facilitation	*Theoretical methods: *Information Facilitation	*Theoretical methods: *Information Facilitation	*Theoretical methods: *Habit formation Social comparisons Mobilising social networks Modelling
	
	*Practical application: *Free FV programme	*Practical application: *Teacher leaflet with ideas for a class- based FV break Provision of a class-kit to all school classes	*Practical application: *Ensure variety in the FV delivery (give local FV providers an order list)	*Practical application: *Provision of class kit Music Candle lights FV break FV host/duty (peer-led)

### Design of intervention programme (step 3 + 4)

Step three included the final decisions about the intervention programme and its components. The matrices guided the selection of theoretical methods and practical applications to achieve each change objective (see Tables 1 and 2 for some examples) [51,59]. In agreement with recommendations for best practice we used theoretical methods from a variety of theories such as social cognitive theory, theories of information processing, health belief model, and the socio-ecological framework [51,60]. Programme activities focused on increasing the adolescents' access to FV in schools, families and sport/youth clubs and on reduction of barriers for making FV available in these setting e.g. affordability, physical access, and time costs.

In step 4 intervention tools were decided and programme material was developed and produced. Table 3 describes the intervention components, the timing of them, the determinants they are intended to target, and their theoretical basis.

**Table 3:** Overview of intervention setting, programme activities, determinants, and theory-based methods in the Boost study

**School class** Educational strategy	**Pupil work book/teacher manual to monthlyguided classroom activities **to be integrated in different school subjects. Main activities: Computer tailoring, statistics on FV intake in school class, how much is a portion?, introduction to the five basic tastes, taste testing of FV, FV research & conference, analysis of commercial for unhealthy food, development of commercial for FV, barriers for eating FV in social situations, FV & the body, FV & the family.	2 assignments per month (September-May). Certain assignments are compulsory	D: Awareness of own FV intake, knowledge of recommended intake levels, taste preferences, media influence, peer influence, social skills, short term outcome expectations, family influence. P: to modify predisposing factors which hinders or facilitates motivation for change (e.g. knowledge, awareness, attitude, perceptions); to improve children's FV preparation skills; to make children aware of what influences their FV preferences; to enable children to obtain FV in different situations and develop skills to ask for FV in a variety of settings; to make children aware of the importance of FV intake for health and well-being.	Behavioural experiments, skills training, role play, modelling, social comparisons, barrier identification, relapse prevention, consciousness raising, and information processing.
**School class and home** Educational strategy	**Boost computer tailoring**. Separate tests for fruit and vegetable intake. Tailored feedback.	September (in class), December (at home) and May (in class)	D: Awareness; taste preferences, situational norms. P: to increase children's awareness of recommended intake and own intake; to change children's perceptions of appropriate time, settings and occasions for eating FV; to change taste preferences; to collect process data on use of FV programme.	Self-monitoring of behaviour and feedback, reinforcement, consciousness raising, and taste acquisition theory.

**School class** Educational strategy	**Script for FV project week/pupil workbook**. Day 1: Set FV goals or make day meal plan with FV, day 2: examine access to FV at home and in local area, day 3: Visit supermarket and make a dish with V, day 4: Boost journalist for a day (interview peers about eating habits), and day 5: Self-evaluation of meeting FV goals/meal plan and school event with parents in the evening.	One week e.g. in October/November. Two days of the programme are compulsory for schools	D: Self-efficacy, situational norms, perceived FV availability, shopping skills, FV preparation skills, parental- and peer influence. P: to modify predisposing factors which hinders or facilitates motivation for change (e.g. knowledge, awareness, attitude, perceptions); to improve children's skills.	Specific goal setting, review of behavioural goals, planning, social comparisons, skills training, information seeking of FV access.

**School** Educational Strategy (training of staff)	**One-day-workshop for year 7 teachers: **1. Information on rationale for Boost and intervention components. 2. How to integrate FV in schools (FV breaks and hands-on teaching). 3. Cooking activities with cook. 4. Teacher feedback on 1st draft of Boost educational material.	Before intervention start (April 2010)	D: Knowledge, attitude, skills for preparing FV snacks. P: to establish motivation and create a receptive environment; to ensure the programme is feasible and acceptable to the teachers and do not increase their workload; to provide inspiration for FV breaks; to facilitate teacher network. Formative research: teacher feedback on preliminary drafts of educational material.	Skills training, prompt identification as role model and social support. Social comparisons. Reinforcement.

**School class** Educational strategy	**3 competitions for pupils from intervention schools **based on curricular activities (Prize: money for school class): 1) Best FV commercial or FV research poster, 2) best map of access to FV in local area, 3) nomination of 3 favourite FVs and 3 least favourite FVs. Boost posters for school and classrooms of year 7 pupils.	Distributed in January 2011 (Spring term). Deadlines: Feb/March and April/May2011	D: Attitude. P: to enthuse children to stay motivated; to create sustained project support. Midterm reminder of curricular component.	Reinforcement, cues/reminders.

**School/home** Educational and environmental strategy	**Parent-school meeting: **information on Boost/launch of the intervention.	Visit by Boost project group in August/September 2010	D: Parental support, parental knowledge, parental attitude. P: to inform parents about the intervention; to establish motivation and create a receptive environment; to make parents understand the value/benefits of the intervention; to create parental support towards intervention goals; to create awareness about recommended intake levels for 13- year-olds and actual intake levels among Danish teenagers. To prompt parents to support their children in eating more FV.	Prompt social support, and consciousness raising.

**School/home** Educational and environmental strategy	**Parent event as part of project week**	One day during project week e.g. in October or November	D: Social norms, social support. P: to create parental support.	Prompt social support

**Home** Educational and environmental strategy	**Guided pupil-parent activities as part of pupil workbook: **examine access to FV at home, examine parents' and siblings' FV taste preferences and monitor parents' intake of FV during one week.	2 assignments during September-June	D: Home availability, family taste preferences, family FV intake, awareness. P: to make children aware of what influences their FV preferences, to prompt children to find FV at home; to make parents aware of own behaviour.	Prompt social support, consciousness raising, environmental change.

**Home** Educational and environmental strategy	**Parental newsletters: **FV snacks, FV throughout the day, FV & sport, barriers for serving FV, acquired taste & taste prejudices, tips to continuance of FV school programmes after funding exceeds, preservation and storage of FV, saving time and money when shopping FV, recipes on quick simplemeals with FV.	6 issues: October, November, January, Marts, April, May	D: Parental facilitation, parental knowledge, attitude, modelling, situational norms, accessibility, perceived parental barriers (perceived affordability, satiety value, preparation methods, time), modelling. P: to make parents aware of own behaviour and act as role models by eating FV with children; to prompt parents to increase availability/accessibility to FV at home; to prompt parents to make FV easy accessible (ready-to-eat) for their children; to prompt parents to provide their children with FV to bring to school.	Prompt identification as role model, social support, barrier identification, and environmental change.

**Local community/school** Educational strategy	**Guided pupil visits in grocery stores **in school neighbourhood (pupil workbook).	One day during project week e.g. in October or November	D: Knowledge, media influence, perceived FVavailability, shopping skills. P: to make children aware of how grocery stores try to influence what people purchase, to modify predisposing factors which hinder or facilitate motivation for change (e.g. knowledge, awareness, attitude, perceptions) as well as skills for shopping FV.	Prompt barrier identification, skills training

**Local community/school** Educational strategy	**Create a map on where to find FV **in school neighbourhood (pupil workbook).	One day during project week e.g. in October or November	D: Perceived FV availability, skills. P: to modify predisposing factors which hinder or facilitate motivation for change (perceptions); to make children identify different places where they can get FV.	Prompt barrier identification

**Local community: sports clubs** Educational and environmental strategy	**Facts sheets to coaches and managers of sport clubs: **1) How to increase children's access to FV during sport (e.g. during practices, competitions, all- day events), 2) to teach children the importance of healthy food when being physical active and 3) to be aware of the coach's status as a role model for eating behaviours.	January (to be implemented in the spring term)	D: Availability, social support, modelling, attitude. P: to increase children's access to FV in leisure time, to encourage sports coaches to be role models.	Provide information about behaviour- health link. Facilitation. Prompt identification as role model, social support, and environmental change

**Local community: youth clubs** Educational and environmental strategy	**Facts sheets to managers of youth clubs: **1) Tips to increase children's access to FV in leisure time (e.g. offering FV snacks, providing recipes with FV), and 2) to teach children the importance of healthy food.	January (to be implemented in the spring term)	D: Availability, social support, knowledge, attitude. P: to increase children's access to FV in leisure time.	Provide information about behaviour- health link. Facilitation. Prompt social support and environmental change

**Local community/home/leisure time:** Educational and environmental strategy	**Newsletter to parents**: tips on healthy, nutritious snacks and beverages for sporty teenagers. Information about recommendations.	January 2011	D: Home facilitated leisure time availability, parental support, parental knowledge, situational norms, and accessibility. P: to prompt parents to provide their children with FV to bring to leisure time activities; to make FV a natural part of leisure time activities.	Provide information about behaviour- health link. Prompt social support and environmental change. Facilitation. Mobilising social networks.

**Local community:** Educational and environmental strategy	**Boost poster **for local providers of FV and participating sports- and youth clubs.	Distributed in January 2011 (Spring term)	D: Attitude, norms, awareness. P: to create project support; to promote social responsibility image of involved partners. Midterm reminder.	Cues/reminders

**Abbreviations: **F = fruit, FV = fruit and vegetables, V = vegetables

### School component

#### One-day workshop for teachers

Before the start of the intervention, principals at all intervention schools were asked to recruit local coordinators among teachers. Their task was to receive and redistribute information concerning the Boost intervention to other teachers and to work as Boost ambassadors. We also asked the principals to recruit two teachers for a teacher workshop prior to the intervention start, preferably the local coordinators. The course was developed in collaboration with the Copenhagen Food House, established by the City of Copenhagen to raise quality of meals offered to citizens - and to create a healthy and happy public food culture. The aim of the workshop was to motivate, inspire and prepare the teachers to implement the Boost intervention (Table 3). The participating teachers were intended to act as change agents and facilitate a receptive environment when returning to their schools.

#### School availability of FV

The key component of the intervention programme was to enhance daily availability through free FV provision to all year 7 pupils at intervention schools. The cooperative owner of a Danish consumer goods retailer chain organised and co-financed the delivery of FV to the schools. In addition the free FV delivery was supported financially by the EU school fruit scheme. We aimed at designing a sustainable, local community-based solution, where the cooperative owner decentralised the delivery of FV by recruiting supermarkets that were located near the intervention schools (local FV providers). WHO recommends eating a variety of FV to ensure an adequate intake of phytonutrients, vitamins, minerals, and dietary fibres [61]. We decided on a monthly basis the kind of FV to be delivered to schools to ensure sufficient variety during the intervention period and equivalent selection across intervention schools. Exposing children to a variety of FV was a priority in this study to challenge and develop children's taste preferences and to ensure that the delivered FV matched many children's taste preferences. Delivery to schools took place twice a week. Accessibility and convenience at school was improved by bringing the FV to the pupils' class desks. Furthermore each school class was provided with a class kit with tools for cutting out FV in order to make the FV appealing and convenient to eat at a daily basis.

#### A pleasant and enjoyable eating environment

The teachers were encouraged to make a FV break in the beginning or end of a class lesson or during a break where the pupils could eat the free FV together with classmates. It was hypothesised that this social practice could change the social and situational norms for eating FV (Table 3). The teachers were encouraged to designate FV hosts/duty among the pupils to be responsible for bringing the FV to the classroom, cutting it up in appealing snacks, serving it to their classmates, and cleaning plates and knives afterwards. By this approach we incorporated an element of peer-led interventions and involved the adolescents actively. Teachers were encouraged to eat FV together with the pupils (role modelling).

#### Boost classroom curricular activities

The educational component in school consisted of a teacher manual, a manual for an optional project week and a pupil workbook including a computer tailoring module. The material was designed to change cognitive factors such as knowledge, skills, attitudes, taste preferences and to encourage pupils to reflect on the FV provided (Table 3). Curricular activities were planned for different subjects (Danish, Maths, Geography, History, Home economics, Physical Education), according to learning goals consistent with the national objectives defined by the Danish education act. The teacher manual included a proposal for a time schedule for when to teach each tutorial to ensure that pupils were exposed to Boost material on a monthly basis.

#### Development of written teaching material

Existing teaching materials from similar intervention projects (the Pro Children study, the HEIA study), the Danish 6 a day campaign and Scandinavian health educational efforts were reviewed to identify tutorials which addressed the selected target determinants [62-68]. Materials were adapted to the study purpose and target group. New tutorials were developed if tutorials were not available or inadequate. A first draft was discussed with two home economics teachers regarding appropriateness for the target group and feasibility for teachers, and revised accordingly. A second draft was presented for teachers from intervention schools at the one-day workshop for their feedback and to stimulate local ownership to the material. A third revised version was reviewed by two teachers at a college for education to estimate the necessary preparation time for teachers for each tutorial and to specify the national learning objectives met by different tutorials. This information was included in the final version of the material. Finally, a graphic designer set up an appealing material.

#### Computer tailored feedback

Computer tailoring appeal to adolescents [39]. The Boost computer tailored feedback messages were tailored to the 13-year-olds' FV intake, awareness levels, taste preferences, and leisure time activities. The Boost computer tailoring program was designed to meet four purposes:

1. Raising awareness of own FV intake. The adolescents were to complete the test three times over the course of the intervention. Their answers were stored in the system, so they could monitor their own intake over time (ipsative feedback).

2. Changing FV taste preferences. The personal feedback suggested recipes to try FV, the 13-year-olds had reported they did not like, in a new way.

3. Changing situational norms for eating FV. The personal feedback message contained ideas to eating FV with friends and at leisure time activities.

4. Process evaluation: The program featured questions about 13-year-olds' usage of the free FV delivered by Boost, including reasons for not eating it. Pupils were asked to complete the program in the beginning, middle and end of the intervention period, favouring a more detailed description of intervention dose. The pupils logged into the computer tailoring module by a personal identification number leaving the possibility to merge the computer tailoring data with data from baseline and follow-ups.

#### Development of Computer tailoring

The Boost computer-tailored program was designed using TailorBuilder version 1.8 developed by OverNite Software Europe BV [69]. The Boost computer-tailored program was based on the Norwegian version of Pro Children computer tailoring for 11-year-olds [64]. Four year 7 pupils (two girls and two boys) were asked to test and comment on the Norwegian version. This version was translated into Danish, adjusted to fit with the older target group and new questions and feedback messages were developed. This Boost version was pilot-tested among five year 7 pupils (three boys and two girls) who participated in a focus group to once more examine comprehension, appeal, layout and the relevance of questions, answer categories and personal feedback. Based on this pilot study a final version was developed and pretested among colleagues before the program was launched at intervention schools.

### Home component

#### Parent school meetings

At the beginning of the intervention period (August/September 2010) the Boost project was presented at parent meetings at 18 intervention schools. The parents were encouraged to support their teenagers in eating FV and to participate in the curricular activities (teenager-parent home assignments). As the parent meetings coincided with the baseline data collection, we left out advice on how to increase FV intake among adolescents to avoid influencing the parents' responses to the baseline questionnaire. The information was later included in a newsletter.

#### Parental newsletters

Parents received six newsletters in the intervention period. The purpose of the parental newsletters was to give ideas of how to serve more FV in their families, as well as to inform parents of the Boost activities in school (Table 3). We used literature on parental barriers for eating and serving FV to adolescents [70-73] and brainstormed on ideas for content with a 6 a day campaign leader [68]. The newsletters were decided to target the following modifiable determinants: home availability (variety), home accessibility (parental facilitation), 13-year-olds' access to FV at sports-and youth clubs, parental knowledge, parental intake and perceived parental barriers for making FV available to children at home (price, time costs, concerns regarding whether FV can satisfy hungry teenagers' appetite, food preparation skills, family taste preferences, and choosiness of teenagers). The newsletters were set up by a graphic designer and pre-tested among colleagues with children in year 7 to ensure the relevance and quality. The local coordinators at each school were asked to post the newsletters at the school's website for parents (parental intranet).

#### School event

As part of the FV project week schools were encouraged to invite parents to experience the on-going Boost curricular activities at an event organised by the 13-year-olds.

#### Teenager-parent assignments

The pupil workbook included assignments to complete at home together with the family. The purpose was to target pupil-perceived availability and stimulate a family discussion about the 13-year-olds' and the family's favourite FV to influence future FV purchases. The assignments were followed up by discussions in class on how family member influence each other's taste preferences and FV intake.

### Local community component

The Boost intervention aimed at reaching 13-year-olds in their leisure time and local community but due to budget and time constraints this intervention component was not as intensive as the school-and home component. Three intervention settings were selected with a focus on increasing perceived and actual availability of FV: 1) local area (e.g. grocery stores), 2) youth clubs, and 3) sports clubs. We originally planned to create a virtual community on Facebook for pupils from all intervention schools with competitions and promotion of FV. However, the idea was abandoned as it posed too many ethical issues e.g. age limits for use of Facebook.

#### Availability in local area

13-year-olds' shopping skills and perceived availability were targeted through the class-based curricular activities. These activities aimed at increasing their attention to e.g. where in their local area they could get FV, which FV were available and to how shops may influence their customers to buy certain foods. Furthermore, the adolescents were trained in analysing means used in adds to change consumer behaviour.

#### Availability of FV in sports-and youth clubs

The intervention in the leisure time was tailored to each intervention school. As part of the baseline survey pupils were asked to provide information on which sports-and youth clubs they attended. Based on this information a total of 18 youth clubs and 21 sports clubs were invited to participate in the second semester of the intervention period. Information sheets were provided to managers of the clubs and coaches of sport teams for 12-14-year-olds. The information sheets encouraged managers to support their members to eat FV during practice/club activities and to make FV available to the adolescents. At the same time a parental newsletter on the same issues were distributed in intervention schools.

### Considerations of at risk groups

School-based interventions have the potential to reach almost every child including socially disadvantaged children. The Boost intervention used a population-based prevention strategy but incorporated features to reach groups with low intake of FV such as boys and adolescents from low-income families, i.e. a free FV programme was chosen over a parental subscription programme [18]. Furthermore, parental newsletters targeted financial barriers for serving FV by 1) providing recipes on quick, cheap, and simple meals with vegetables and 2) highlighting how to save money by using FV in season or frozen and tinned FV. To reach boys we targeted sports clubs.

### Control group

Year 7 pupils, parents and principals in the control group will complete the surveys only. The control schools were encouraged to continue the school year in the intervention period as originally planned before being contacted by our project and not initiate any new FV promoting initiatives besides those planned already. After 1^st ^follow-up surveys were completed, an electronic version of the teaching material was disseminated to the control schools. To ensure sufficient exposure contrast at 2^nd ^follow-up the material could be used in year 7 only and not among our target group who now attended year 8.

### Adoption, implementation and sustainability (step 5)

Adoption and implementation was planned systematically but the time schedule did not allow for creating matrices for change objectives for programme adoption and implementation as recommended by the Intervention Mapping protocol [51].

### Adoption

#### Recruitment of schools

The recruitment of schools was thoroughly prepared. We applied the planning tool 'double entry book-keeping of organisational change' [74] which can be used to analyse stakeholders' resistance to change. We composed a table of four columns for each stakeholder (principals, teachers, parents and schoolchildren) hypothesising downsides (1) and upsides (2) of the current situation before implementation of the intervention as well as possible downsides (3) and upsides (4) after the intervention had been implemented. The information in the first column can be used to establish a sense of urgency with adopters. Columns 2 and 3 represent'the cost of change' to the stakeholders and therefore plausible resistance to project-related change. The fourth column represents the project group's 'vision of a new tomorrow'. The research groups will typically be driven by the positive change expected (from 1 to 4) whereas adopters will be focused on the change from current positive factors (2) to future downsides and loss of benefits (3). This tool makes explicit the critique and resistance to change which you need to take into account in the phase of adoption and implementation [74]. The information from the above process was used to create a speech manuscript (for telephone recruitment) and recruitment material for school management, teachers, parent boards and pupil councils, explaining how the programme would benefit the school.

#### Recruitment of FV providers and fundraising

In approaching the cooperative owner of the consumer goods retailer chain to suggest funding and delivery of FV, we highlighted how their organisation would benefit from participation e.g. gain insight into schools as a market, demonstrate corporate social responsibility and strengthen company image. We outlined the logistics necessitated by the project e.g. frequency of delivery and storage issues. The cooperative owner was responsible for recruiting the local FV providers for all intervention schools.

#### Adoption among teachers and parents

The one-day teacher workshop and the parent meeting intended to engage teachers and parents in the intervention. Local coordinators were intended to function as change agents at their school i.e. motivating the other teachers.

#### Recruitment of sports-and youth clubs

Sports-and youth clubs were automatically included if they met the inclusion criteria. Managers and coaches were informed about their clubs being selected as intervention sites in an email with an electronic baseline questionnaire attached.

#### Planning for implementation and sustainability

The Boost intervention was implemented from September 2010 to May 2011. Intervention providers included teachers, parents, local FV providers, pupils, coaches and managers in sports-and youth clubs, supported by principals, the super market chain, Copenhagen Food house, and the Boost project group. The intervention was designed to be implemented primarily by teachers with minimal help and contact from the research team in order to create a sustainable intervention. All teachers were meant to share the responsibility for implementing the Boost curriculum and FV scheme, supported by the teacher(s) coordinating the project at school level. In case of problems with the quantity or quality of FV delivered to schools, teachers were requested to address the local provider to solve issues of concern. The coaches in the sports clubs and the managers of the youth clubs were intended to facilitate increased access to FV in the 13-year-olds' leisure time. Implementation manuals for teachers, pupils, and local FV providers were developed and distributed (teacher manual for curricular activities, teacher manual on conducting school FV breaks, manual on delivery of school FV for schools and local providers, posters for cutting up FV snacks and hygiene rules for the school class room). Parental newsletters and club factsheets were used to guide parents and club staff in supporting and promoting 13-year-old's FV consumption.

#### Planning for adoption and dose received among teenagers

The following strategies were applied to ensure the pupils' active engagement in the Boost project: Appealing name, visual identity and pupil workbook, interactive learning i.e. hands on-experiments, computer-based assignments such as computer tailoring, competitions, Boost posters distributed for use in the classroom, time for FV breaks, delivery of class kit with an iPod docking station, and the FV hosting/duty.

### Evaluation design (step 6)

Effectiveness of the intervention will be evaluated employing a cluster-randomised controlled trial design with baseline measurements among pupils, parents and school principals in the beginning of the school year (August 2010), and 1^st ^and 2^nd ^follow-up measurements at the end of the school year (May/June 2011) and one year after (May/June 2012).

#### Trial registration

Current Controlled Trials ISRCTN11666034.

#### Power calculations

To assess the adequate sample size of schools and pupils needed to detect a 20% difference between intervention-and control schools in intake of FV at follow-up, we calculated the power of a two-sample *t*-test using the SAS computer code suggested by Donner & Klar (1996) [75]. We based intra-class correlations (= 0.01) on Pro Children data. If 20 schools with two school classes of 20 pupils each were randomly assigned to each intervention group, the analyses showed that there would be more than 90% power to detect a 20% increase in pupils' mean intake of FV between intervention-and control schools (N schools = 40, N pupils = 1,600).

#### Study design, sampling, selection criteria, recruitment, and randomisation

**Figure 2:**
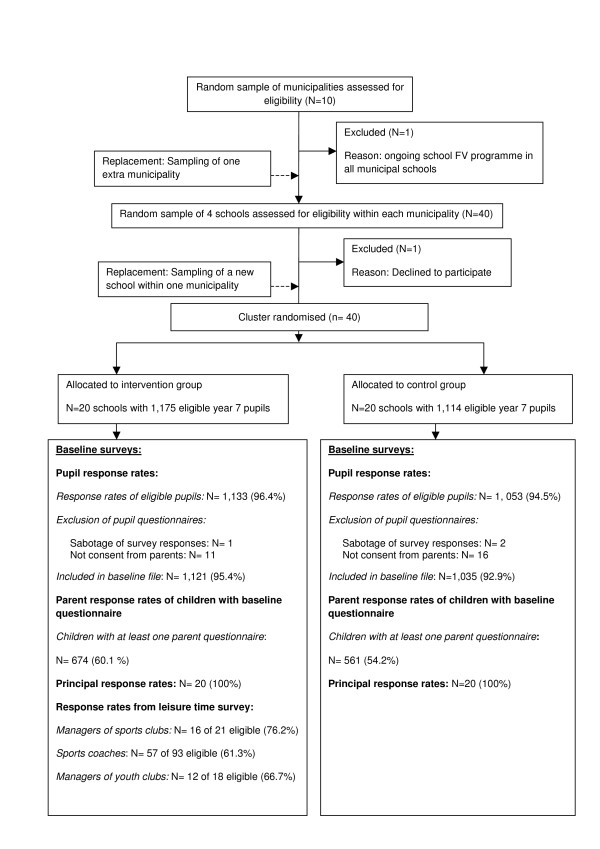
**Flow diagram of sampling, recruitment, randomisation and participation in the Boost study**.

Figure 2 shows the flow of the sampling process. We decided on a study design with ten municipalities with two intervention-and two control schools within each municipality. By this study design we aimed at making intervention-and control schools comparable as schools within the same municipality are exposed to the same political context (local health policies and political standpoints). The inclusion of ten municipalities only also reduced travel costs and made fieldwork feasible (e.g. data collection and thorough process evaluation at schools). A simple two-stage cluster sampling process was applied beginning with a random sample of municipalities (sampling frame: list of all 98 municipalities in Denmark, exclusion criteria: Less than four public schools), followed by a random sample of municipal schools (sampling frame: lists of all schools in selected municipalities). Eligible schools had minimum two school classes of year 7 pupils, exclusion criteria: special needs schools, and private schools. Using a computer-generated list of random numbers we randomly ranked Danish municipalities and the schools within them. The first ten municipalities from the randomly ordered municipal list were selected and within each of them the first four schools from the randomly ordered school lists were drawn. By telephone, principals were invited to take part in the study. They received a letter with factsheets addressing the school administration, teachers, parent board, and pupil council immediately after the telephone conversation. Schools accepting to participate were asked to sign a written contract disregarding the result of the randomisation. In one of the included municipalities a school refused to participate due to involvement in too many projects and was replaced by a new school from the list. To ensure sufficient exposure contrast, one of the sampled municipalities in which schools already had a free FV programme, was excluded. A random sample of schools from the next municipality on the list of randomly selected municipalities was approached and all schools contacted in this municipality accepted to participate.

On receipt of a signed contract on participation from all four schools within each municipality, schools were randomly allocated into intervention-and control groups by lot. All school classes of year 7 were asked to participate. School classes of children with special needs were excluded from the evaluation study due to the complexity of the questionnaire.

### Effect-evaluation

#### Questionnaires

All Boost questionnaires were based on the validated Pro Children questionnaires for pupils, parents and staff, supplemented with items from other studies (e.g. The HBSC study, the HEIA study, North Carolina Child Health Assessment and Monitoring Program Survey (CHAMP) either transferred without any revision or adapted to the Boost study [56,76-79]. We also developed new items specifically related to the Boost study. The pupil baseline questionnaire was tested among year 7 pupils (3 girls and 3 boys) to test time range for answering, followed by a focus group interview about comprehensiveness, layout etc. The questionnaire was changed accordingly. Questionnaires for parents, principals and representatives were pretested among colleagues and friends, prioritising parents of year 7 pupils. Table 4 summarises the key measures included in the Boost surveys matching the programme theory (Figure 1).

**Table 4:** Main concepts and measures in the Boost study

Concepts	Operationalization/definition	Pupil Parent Principal			Teacher (PE)*	Observations/photos	Local provider (PE)*	Manager youth club*	Manager sports club*	Coach sports club*	FG* (PE)	CT* (PE)
Distal outcome**:												

Adolescents' FV intake (amount)	24-hour recall questionnaire (converted to grams per day)	X										

Adolescents' usual FV intake	FFQ	X										

Proximal outcomes**:												

ENVIRONMENTAL DETERMINANTS												

Actual and perceived availability:	Covered dimensions: quantity, variety, quality, appearance, accessibility, convenience, parental facilitation, visibility, time for eating FV, access to unhealthy food											

*at school*	(see dimensions above)	X	X	X	X	X	X				X	X

*at home*	(see dimensions above)	X	X									

*in the leisure time*	(see dimensions above)	X	X					X	X	X		

*in the (school-) neighbourhood*	(see dimensions above)	X	X	X		X						

SOCIAL DETERMINANTS												

Social norms: situational norms	Number of meals, situations, occasions and settings perceived as appropriate for eating FV	X	X									

Parental influence												

Dietary knowledge	Knowledge of national FV recommendations		X									

Attitude	Perceived importance of child eating FV		X									

Actual and perceived FV intake	FFQ/24-hour recall questionnaire similar to distal outcome	X	X									

Usual intake of unhealthy food	Intake of soft drinks, sweets and salty snacks (FFQ)		X									

Modelling	If parents are eating FV together with children	X	X									

Perceived barriers	Access, time, money, convenience, preparation skills		X									

Peer influence												

FV intake	If best friends are eating FV	X										

Social norms: peer norms	If it is 'cool' to eat FV in school class. If the majority of peers are eating FV in school and in the leisure time	X									X	

Teacher influence												

Modelling	How often teachers eat delivered FV together with pupils				X						X	

Attitude	Perceived importance of promoting healthy eating in school				X						X	

Sport coach influence												

Modelling	If sports coaches are eating FV during practice							X				

Attitude	Perceived importance of children eating FV during practice							X				

Proximal outcomes**:												

PERSONAL DETERMINANTS												

Taste preferences	Liking of FV and number of different FV liked	X										X

Dietary knowledge	Knowledge of national FV recommendations	X										

Awareness	Awareness of whether they meet national recommendations	X										X

Short term outcome expectations	Perceived immediate effects of eating FV: e.g. more energy, feel better, FV not filling enough, FV allergy	X										

**Potential positive side effects:**											X	

More energy	Loss of energy during the school day	X			X						X	

School concentration	Unable to concentrate during class lessons	X			X						X	

Wellbeing	Children's level of life satisfaction, if they most of the time are feeling well and full of energy	X			X							

Usual intake of unhealthy food	Intake of soft drinks, sweets and salty snacks (FFQ)	X										

School: environmental changes	Nutrition policies, FV programme, food availability		X			X						

**Unintended adverse outcome:**											X	

Bullying	How often they have been bullied at school in the past couple of months	X										

Weight- and eating related teasing	If class mates are teasing each other because of weight, lunch bags or eating habits	X										

School class thriving	If pupils in their class enjoy being together	X										

Free FV replace FV eaten at other times of the day**	Eating less FV at home (24-hour recall questionnaire). Fewer children bringing FV to school from home	X	X								X	

**Moderators:**												

Socioeconomic position	Parent occupational social class	X	X									

Socioeconomic position	Parental education		X									

Gender	Boys and girls	X										

Implementation degree	Dose delivered/received, fidelity: adherence to protocol	X	X	X	X	X	X	X	X	X	X	X

**Abbreviations: **CT = Computer tailoring, FG = focus groups with children and/or teachers, FV = fruit and vegetables, PE = process evaluation, * at intervention schools only, **measured for fruit and vegetables separately

#### Measures

The primary outcomes measures were corresponding to the distal outcomes in the programme theory and measured by two pupil-reported measures at all three time points:

*Total fruit-, vegetable-and FV intake in grams per day *(in school + outside school) measured by the validated Pro Children pre-coded 24-hour recall questionnaire [77]. The 24-hour recall questionnaire measured how many pieces or portions of specific types of FV the pupils had consumed in five different time intervals on the previous day: before school, at school, after school, at dinner and after dinner. Pieces and portions of FV were converted into grams based on food weights applied by experts in the field [77,80]. The vegetable measure excluded potatoes. The fruit measure included 100% natural juice but juice was only counted as one portion/100 grams a day regardless of the number of glasses consumed. **Pre-specified success indicator: **Based on the effect size obtained in the Pro Children study [21], we aimed at a 20% increase in FV intake at intervention schools at 1^st ^follow-up versus baseline and control school. This increase should be maintained into 2^nd ^follow-up.

*Habitual fruit-, vegetable-, and FV intake *was measured by the validated Pro Children food frequency questionnaire (FFQ) [77]. Fruit intake was measured by one item on fresh fruit. Vegetable intake was measured by three separate items: salad or grated vegetables, other raw vegetables, and cooked vegetables. **Pre-specified success indicator: **Significantly higher proportions of pupils eating FV daily at intervention schools at 1^st ^follow-up. This increase should be maintained into 2^nd ^follow-up.

Secondary outcomes were measured at all three time points and included 1) targeted determinants, corresponding to the proximal outcomes in the programme theory such as taste preferences, availability, and perceived parental barriers for serving FV at home and explorative outcomes, corresponding to intended and unintended side-effects such as wellbeing and eating-related teasing,

#### Proximal outcomes of the intervention: Changes in determinants

Environmental, social and personal determinants were measured by questionnaires to pupils, parents, principals and representatives from sports-and youth clubs. The intervention programme was designed to create significant, positive changes in specific determinants of teenagers' FV consumption at intervention schools at 1^st ^follow-up versus baseline and control schools. As shown in Table 4 the Boost study is characterised by thorough measures of both perceived and actual social and environmental influences (e.g. parental intake and home availability) using different data sources. We also measured potential confounders of the association between intervention and FV intake (determinants of FV intake which the intervention was not tailored to) such as school nutritional policies, family meal habits, family structure, and ethnic background (not shown in Table 4). Parental SEP (moderator in Figure 1) will be based on questions to parents and pupils on each parent's educational and occupational background.

*Predefined explorative secondary outcomes: changes in positive and negative intended and unintended side-effects*: Intended positive side effects and unintended adverse effects will be measured by questionnaires to the pupils and parents. We will assess whether there are significant changes in these among pupils at intervention schools versus baseline and control group.

Intended positive side-effects: At follow-up, the increased access to FV at intervention schools was expected to significantly decrease proportions of pupils at intervention schools who felt out of energy, had difficulties in concentrating during class in school and who ate unhealthy food almost daily. Also, we expected significant positive changes in pupils' well-being. By use of questionnaires to the school principals, we will measure whether participation in the Boost project has initiated some environmental changes and capacity building at the intervention schools at 2^nd ^follow-up (e.g. initiation of FV programmes, increased sale of FV in schools, increased focus on school food policies or limiting pupils' access to unhealthy food at school).

Unintended adverse effects: It will be evaluated whether the intervention and the increased focus on healthy eating has produced a significant increase in weight-and eating-related teasing or bullying. There is a risk that the free FV in school only replace FV eaten at other times of the day which will hinder an effect on the pupils' total intake of FV [22]. We will therefore analyse whether fewer adolescents eat FV at home and bring FV to school from home after the intervention. As unintended adverse effects are difficult to predict they will also be illuminated through qualitative interviews with pupils and teachers as part of process evaluation (see Table 4).

#### Planned statistical effect analyses

The primary aim of the effect evaluation will be to assess whether our goal of a 20% increase in FV intake has been achieved. All further impacts of the intervention such as intended proximal effects (determinants) and unintended positive and negative side effects will also be subject to evaluation. Outcomes will be analysed after the principle of intention-to-treat including all pupils in the arm to which they were allocated whether or not they received (or completed) the intervention given to that arm. These results will be compared to per protocol analyses of pupils at schools that complied sufficiently with the intervention manual to exhibit the effect of treatment at the individual level. Descriptive analyses will be used to calculate the net effect of the intervention ((follow-up intake_intervention _-baseline intake_intervention_) -(follow-up intake_control_-baseline intake_control_)). The effect of the multicomponent intervention will be analysed for each and all six primary outcome measures of FV intake. To account for the cluster-design and repeated measurements, changes in consumption from baseline to first-and second follow-up will be analysed by multilevel multivariate regression analyses. All analyses will be stratified by gender and we will examine if the intervention have differential effects on FV intake among pupils from low and high social class. Our hypothesis is that the intake of teenagers from low income families at school will benefit more from the free FV provision than their peers from high income families as they may not usually bring FV from home.

In another analysis implementation degree (high/medium/low) will be included as we hypothesise that adolescents at schools/school classes who have been exposed to a large dose of the intervention will have a higher intake at 1^st ^follow-up compared to adolescents at schools where the intervention has not been fully implemented.

In a third analysis we will try to examine if it is possible to decompose the effects of the different intervention components through mediation analysis. We will analyse the effect of the different intervention components on the determinants (proximal outcomes) they have been tailored to. One hypothesis is for example that pupils at intervention schools will like a larger variety of FV than the pupils at control schools at 1^st ^follow-up due to the free FV provision. In a second analytical step we will examine if change in intake is preceded by change in determinants through mediation analyses.

Parent non-response at baseline and pupil and parent attrition between baseline and 1^st ^and 2^nd ^follow-up will be analysed by multilevel analyses using data from pupil baseline survey and national registries.

#### Baseline data collection

Pupils completed internet-based questionnaires in class and brought home questionnaires for their parents to complete. As fathers and mothers may influence their children's food habits differently [81], both parents were invited to complete questionnaires. At all schools, trained project staff helped teachers carry out the surveys. The year 7 pupils received standardised oral instructions and a factsheet with definitions and photos of portion sizes. During the baseline visit, observations and photo documentation were made of the school food environment such as presence of canteen or fruit tuck shop, and classroom conditions. A pupil or teacher in each class was briefly interviewed about lunch habits among classmates and unwritten school rules. All principals completed an electronic questionnaire on structural, physical and social characteristics of their school. Pupil, parent and principal surveys and observations will be repeated at 1^st ^and 2^nd ^follow-up.

In January 2011, managers and coaches of clubs and teams were asked to complete internet-based baseline surveys on availability of FV and unhealthy food and existence of policies before receiving Boost information material. A 1^st ^follow-up was carried out in September/October 2011.

#### Response rates and characteristics of pupils at baseline

As shown in Figure 2, response rates were high (> 94%) among both pupils and school principals at intervention-and control schools. A few pupil questionnaires were excluded from the baseline data file because of lack of parental consent. Only three pupil questionnaires were unserious and therefore excluded. The parental response rate was lower in both groups, but higher at intervention schools. Most of the returned parent questionnaires (60%) were completed by female caretakers. The response rates among coaches and managers of sports and youth-clubs were good (> 60%).

Baseline characteristics of pupils from intervention-and control schools were compared by chi-square tests for proportions and *t*-tests for continuous variables to verify the adequacy of the randomisation procedure. The baseline data file included 1,121 pupils from intervention schools and 1,035 pupils from control schools. There were no significant socio-demographic and outcome differences between intervention-and control groups at baseline except for a significant larger percentage of pupils at control schools reporting their parents to be born in Denmark (Table 5). Forty percent of the pupils reached the WHO recommendations of daily FV intake and one fifth accomplished the national recommendations. Less than half of the pupils consumed fruit daily and only one fifth of the pupils consumed raw vegetables daily. The proportions of pupils having access to FV at home, in school and leisure time were almost similar in the two samples at baseline (Table 6). One fifth of the pupils did not attend a sports-or youth club.

**Table 5:** Baseline characteristics of pupils at intervention-and control schools: socio-demographics and primary outcome measures (significant differences are highlighted in bold, p < 0.05)

	Intervention sample (N = 1,121)		Control sample (N = 1,035)		
**Sociodemographics**	**Percentage (N) of pupils reporting the given characteristic**	**N Missing**	**Percentage (N) of pupils reporting the given characteristic**	**N Missing**	**P-value from chi-square test or *t*-test**

Boys	51.8% (581)	0	51.9% (537)	0	p = 0.980

Mother is unemployed*	12.0% (134)	6	10.5% (108)	1	p = 0.169

Father is unemployed*	6.8% (73)	6	5.3% (53)	1	p = 0.237

Mothers' SEP*	26.7% (295)	6	27.9% (286)	2	p = 0.198
High (I + II)	26.7% (295)		29.9% (307)		
Medium (III + IV)	23.9% (264)		21.8% (223)		
Low (V + 7)	22.7% (251)		20.4% (209)		
Other (6 + 8)					

Fathers' SEP*	20.1% (218)	6	18.9% (189)	2	p = 0.319
High (I + II)	37.8% (411)		41.4% (415)		
Medium (III + IV)	18.9% (205)		16.7% (167)		
Low (V + 7)	23.2% (252)		23.0% (230)		
Other (6 + 8)					

Pupil was born in Denmark	95.1% (1060)	6	95.0% (983)	0	p = 0.988

Mother was born in Denmark	**83.4% (930)**	6	**86.1% (891)**	0	**p = 0.035**

Father was born in Denmark	**84.1% (938)**	6	**88.3% (914)**	0	**p = 0.006**

Speaking Danish at home	95.7% (1068)	6	96.2% (996)	0	p = 0.597

**Primary outcomes**					

Mean FJ intake (SD)**	232.3 g/day (197.3)	3	226.3 g/day (195.8)	0	p = 0.512

Mean V intake (SD)**	129.5 g/day (158.0)	3	142.1 g/day (165.5)	0	p = 0.094

Mean FJV intake (SD)**	361.8 g/day (262.4)	3	368.4 g/day (262.7)	0	p = 0.588

Meeting WHO guidelines of eating ≥ 400 g of FJV daily (24-hour recall)**	40.7% (395)	3	43.5% (395)	0	p = 0.818

Meeting national guidelines of eating ≥ 600 g of FJV daily (24-hour recall)**	21.1% (205)	3	22.1% (201)	0	p = 0.670

Eating F daily (FFQ)	48.2% (538)	5	48.5% (502)	0	p = 0.891

Eating raw V daily (FFQ)	20.3% (227)	5	21.5% (223)	0	p = 0.492

Eating cooked V daily (FFQ)	6.3% (70)	5	5.7% (59)	0	p = 0.577

**Abbreviations: **FJ = fruit including maximum one portion of 100% natural juice, FJV = fruit including maximum one portion of 100% natural juice plus vegetables, FV = fruit and vegetables, SEP = socioeconomic position, V = vegetables. * Exclusion of pupils who do not have/see mother or father, N = 79 (Intervention sample: mother: N = 10, father: N = 29. Control sample: mother: N = 8, father: N = 32). ** Exclusion of over-eaters defined as pupils eating > 1,000 gram FJV. N over-eaters = 274 (Intervention sample: N = 148, control sample: N = 126).

**Table 6:** Baseline characteristics of pupils at intervention-and control schools: key proximal outcomes

	Intervention sample (N = 1,121)		Control sample (N = 1,035)		
	**Percentage (N) of pupils reporting the given characteristic**	**N Missing**	**Percentage (N) of pupils reporting the given characteristic**	**N Missing**	**P-value from chi-square test**

**School availability of FV**					

Having daily access to F in schools	50% (558)	6	46.6% (480)	4	p = 0.106

Having daily access to V in schools	26.9% (300)	6	27.4% (282)	4	p = 0.816

**Home availability of FV**					

Having daily access to F at home	46.8% (522)	6	43.4% (448)	2	p = 0.109

Having daily access to V at home	53.8% (600)	6	55.8% (576)	2	p = 0.365

**Availability of FV at leisure time activities**					

Always having access to F in at least one sports- or youth club*	25.6% (232)	8	30.0% (245)	9	p = 0.047

Always having access to V in at least one sports- or youth club*	13.0% (118)	8	12.0% (98)	9	p = 0.503

**Abbreviations: **F = fruit, FV = fruit and vegetables, V = vegetables. *415 pupils who did not attend sports-or youth clubs were excluded from the analyses (Intervention sample: N = 207, control sample: N = 208)

### Process evaluation

The implementation of the intervention has been monitored by a thorough process evaluation throughout the entire intervention period. The purpose of this evaluation was 1) to evaluate the recruitment of schools (barriers and facilitators of participation), 2) to identify barriers and facilitators for the implementation, 3) to collect information on the intervention dose delivered by the teachers and received by the pupils and on coverage: did it reach all pupils irrespective of e.g. gender and socio-economic position, 4) to monitor intervention fidelity (adherence to implementation manuals), 5) to assess acceptability of the intervention, 6) to be able to decompose effects of each of the multiple components of intervention, and 7) to be able to provide recommendations on how to organise future school FV programmes [32,33]. Process evaluation questions were guided by Linnan & Steckler's conceptual framework, Rogers' diffusion of innovation theory, implementation theory and other process evaluation studies [31,33,39,82,83]. Evaluation methods included teacher logbooks, computer tailoring track records, participant observations, focus group discussions with teachers and pupils, telephone interviews with local FV providers, and surveys among pupils, teachers, parents, and principals. Thorough description of the process evaluation is beyond the scope of this paper.

#### Contamination issues

Using data from 1st follow-up questionnaires among pupils and principals it will be measured 1) if any environmental or educational activities have been conducted at control schools to promote FV consumption during the intervention period, 2) if the intervention in schools, sports-and youth clubs have spilled over to the control groups, 3) if any interfering events have influenced the implementation and effectiveness of the intervention e.g. schools closing down or change of principal or teachers.

### Economic evaluation

Resources used for the intervention (training, production and implementation costs of intervention material and unit price and delivery expenses of FV, teacher preparation time, extra cleaning because of FV programme etc.) have been systematically registered for cost-consequence analysis [84]. A societal perspective will be applied including analysis of the societal cost of nationwide roll-out of the Boost programme. These calculations may assist policy makers, principal and other stakeholders in evidence-based resource allocation by informing them about affordable, effective dietary interventions.

### Ethical considerations

The Boost Project adheres to all Danish ethical standards and has been approved by the Danish data protection agency (J.nr. 2010-54-0974). When schools were invited to participate written information was sent to principals, parent boards and pupil councils at all schools explaining the implications of participating in the study. For focus groups and surveys, all respondents were informed that participation was voluntary and anonymous and that all data would be handled confidentially. Parents could ask the research group to exclude their child's survey responses from the database by ticking a box on the front page of the parent questionnaire. Furthermore, the parents could ask the research group by email or telephone not to collect the civil registration number of their child. This was collected by the project group to enable register-based follow-up evaluation of long-term effects.

## Discussion

The Boost baseline results confirm the need for interventions as only 40% of the adolescents in this study reach the levels of FV intake recommended by WHO. Likewise, large proportions of pupils in the Boost study reported low access to fruit and especially vegetables in school, home and sports-and youth clubs at baseline.

The novelty of the Boost intervention is that it is one of the first large-scale, school-based, multi-component, multi-level interventions to be implemented for a whole school-year and evaluated with long follow-up and in a randomised controlled design in Denmark. It is based on comprehensive reviews of both quantitative and qualitative studies of factors which promote teenagers' intake of FV [3,6], it focuses on 13-year-olds, it targets both fruit and vegetable consumption, it has a strong focus on availability in all settings relevant to teenagers, it implements a free FV programme in a Danish context in combination with educational components, it focuses on changing norms/school class environment/social practise around eating FV, and it explores ways to include sport-and youth clubs in health promotion. The survey instruments measure groups with low intake of FV such as boys and low social class, leaving opportunity for analysis of effect modification. The project tests innovative constructs such as perceived appropriateness of eating FV in different occasions, settings and situations and a multidimensional measure of availability at home and in schools [6]. The thorough process evaluation based on multiple methods and data sources will give a comprehensive picture of characteristics of the intervention and the school (context and composition) influencing implementation of educational and environmental strategies in families, schools and sports-and youth clubs. The study design enables us to examine which methods are efficient in changing specific determinants as we may link changes in determinants to whether the specific components were effectively administered. Successful recruitment of schools and high response rates among pupils have resulted in the desirable number of schools and pupils as estimated in the power calculations. We have succeeded in establishing randomisation. Analysis of baseline data showed almost equivalent groups when considering socio-demographic characteristics, primary outcomes, and FV availability at school, home and in sports-and youth clubs, a good starting point for estimating effects of the intervention at 1^st ^and 2^nd ^follow-up. In spite of the random allocation of schools to intervention-and control groups, significantly larger proportions of pupils at control schools had parents born in Denmark which must be taken into account in the effect analyses.

### Comparison with other studies

#### Planning process

As many previous intervention projects the Boost intervention has been designed using the systematic theory-and evidence based planning tool Intervention Mapping [51,56,57,85]. In addition we tested some new planning strategies in the study: 1) To ensure ownership of the Boost curriculum and the applicability of the teaching material in intervention schools, we gathered teachers from all intervention schools to comment on a preliminary draft of the teaching material at a one-day workshop before intervention start. 2) Many countries experience a decrease in the number of schools willing to participate in research projects which compromises the power and representativeness of the studies [86,87]. The recruitment of schools for the Boost study was thoroughly prepared and inspired by the principle of double-entry bookkeeping of organisational change [74]. We believe that these careful considerations about stakeholders' interests together with the schools' prospects of getting FV for free throughout the school year contributed to the successful recruitment process.

#### Multi-component design

Other school-based multi-component interventions have also included environmental strategies to increase access to FV in schools, a school curriculum and the involvement of parents [19,37,57,88]. The Boost study differs from these interventions by involving an older age group of children, by targeting FV intake separately, by serving FV for free and each day throughout the school year, and by adding a new component in youth-and sports clubs which aims at increasing teenagers' access to FV in the leisure time setting. The Boost study has an explicit focus on accessibility in school. Accessibility is increased by means of free delivery, cutting up FV as snacks, and by allocating time for eating FV. For the school curriculum, we built on Norwegian experiences [56,89] and developed a teaching material which was compatible with the official national learning objectives. Unlike most other projects [57,89], we developed tutorials which could be implemented in many subjects instead of only as part of home economics classes. The Boost computer tailoring instrument is a further development of the Pro Children computer tailoring [64]. As a new feature the children's data entries for each time they used the program were stored in the program's memory so the children could monitor the development in their intake over the school year to further stimulate their awareness.

#### Implementation

As in the Pro Children study and the Dutch Schoolgruiten project [57,90], the Boost intervention was designed to be implemented primarily by teachers. In the former studies the responsibility for implementation relied on one teacher alone either the main teacher or home economics teacher. In the Boost study, all teachers of year 7 shared the responsibility of implementing the curriculum and FV scheme, supported by a coordinator at the school. Some studies indicate that peer-led health intervention strategies are effective among teenagers [91-93]. In the Boost study, the pupils are widely responsible for implementing the FV break.

#### Evaluation design

The Boost study differs from other similar interventions by using a random sample of municipalities and schools including all year 7 school classes at each participating school and not only a self-selected sample of school classes, by having larger number of participating schools and pupils in each intervention group, or by randomly assigning schools to intervention group [12,13]. Other studies have included process measures [25,39,89]. We contribute to this field by developing and applying a theory-and evidence based process evaluation model, and by using both qualitative and quantitative methods and different data sources [33,51]. This will enable us to make a systematic, thorough evaluation of the implementation of each intervention component. Items on usage of the school FV programme and motives and barriers for usage have been incorporated in the computer tailoring module to monitor the pupils' access to and usage of the FV programme throughout the school year thereby supplementing information from the baseline and 1st follow-up questionnaires. These data can be merged with the other dataset by the pupils' unique identification number. Together with the measurements of determinants of FV intake (proximal outcomes of the intervention), the process evaluation data will guide the decomposition of effects of each intervention component. As in the Pro Children project [55] we used source triangulation to measure availability in home, school and the local area. Whereas availability in the leisure time was only measured by questionnaires to pupils and parents in the Pro Children study, the Boost study also measured it by questionnaires to coaches and managers of sports-and youth clubs although only in the intervention group. Finally, the project group decided the selection of FV to be delivered to the schools throughout the school year which means that the dose and types of FV delivered to schools are well described.

### Strength and limitations of the Boost study

Merits of the Boost study are 1) the successfully randomised controlled design, 2) the random sample of municipalities and schools, 3) the high response rates and large sample sizes of schools and pupils, 4) the systematic theory-and evidence-based planning procedure of the study, 5) the thorough and comprehensive process evaluation, 6) the triangulation of sources, methods and theories, 7) the use of two outcome measures for FV intake (habitual intake and amount), 8) the participatory approach (inclusion of the teachers in the planning phase), and 9) the measurements of proximal outcomes (determinants of FV intake).

Limitations of the Boost intervention are that outcome measures are based on self-reports, lower response rates among parents, the risk of contamination as some pupils from control schools may attend the intervention youth-and sports clubs and the lack of control group for the leisure time component. Municipalities and schools were randomly selected. High response rates among principals and pupils at baseline minimised the risk of selection bias. However, when we analyse matched teenager-parent data, selection bias may be introduced as a consequence of lower parental response rates.

The FFQ and 24-hour recall measurements were based on the validated Pro Children instrument [77]. The dietary data are based on self-reports and may be over-or underestimated depending on recall bias and social desirability bias. To prevent social desirability bias among pupils we introduced the survey by telling them that it was not an exam and that there were no right or wrong answers. We encouraged them to answer as honest as possible. Most measurements of determinants and sociodemographic factors in the Boost study were based on the validated Pro Children questionnaires [76] and HBSC questionnaires [78,94]. New items were also developed some of which are not fully validated.

Each intervention tool was designed to change specific determinants of FV intake. By means of addressing these determinants as proximal outcomes related to each of the components of the intervention, we may be able to decompose the effects of the multi-component intervention, so that the effect of each of these dimensions becomes apparent. As pupil-and parent questionnaires had to cover many determinants and included a detailed 24-hour recall questionnaire, we had to include only one or few measurements of each of these determinants to keep the questionnaire short. This may compromise the precision of each item and may introduce information bias. As the intervention targets availability we prioritised to have detailed measurements of this multidimensional concept to capture the influences of different aspects of availability (visibility, accessibility, variety, quantity, quality), which is a unique feature of this study.

We adhered to the Intervention Mapping protocol and addressed modifiable and important determinants such as availability and taste preferences. We did not address genetic factors such as bitterness taste sensitivity as they are non-modifiable and the findings from research of the importance of bitter taste sensitivity for FV intake and taste preferences among children and adults are inconclusive [95-99].

We agree with other researchers that it is a time consuming task to follow the Intervention Mapping protocol [57,100] but we found the effort worthwhile. The Intervention Mapping protocol distinguish itself by guiding researchers to make well-considered, explicit theory-and evidence-based decisions in every step of the process of intervention development, implementation, and evaluation [51]. We did not have time to create matrices on adoption and implementation which might have contributed with valuable insights on how to support teachers in implementing the interventions at schools. Creation of these matrices post intervention may qualify the process evaluation. Finally, the sustainability of the Boost intervention is compromised by the project groups' active involvement in the FV programme. We were responsible for the selection of FV delivered to the schools each month. Our intention was to ensure that year 7 pupils at all intervention schools were exposed to a variety of FV, preferably organic, Danish and in season. The downside of this approach is that nobody takes on this task after the termination of the research project at the schools.

### Implications for research

It remains a challenge to ensure sufficient implementation when intervening in schools. The comprehensive examination of implementation processes, barriers and facilitators in the Boost study may contribute with important insights on how to promote implementation of school-based interventions. The systematic and theory-based approach to process evaluation taken in this study may guide development of future process evaluations in intervention studies and yield important knowledge. The incorporation of a data collection in the computer tailoring module is tested in our study and may be a promising tool to inform future process and effect evaluations. The Boost study will provide answers to whether it is possible to disentangle the effects of different components by analysing effects on proximal outcomes (determinants) and will attempt to monitor the dose received of each intervention component. Also the separation of fruit and vegetables as different outcomes may illuminate whether the strategy of targeting fruit and vegetables in the same intervention is an effective way to increase both fruit and vegetable intake. The detailed 24-hour recall data collected at all three time points enable analyses of changes in intake of specific types of FV. Finally, intake of specific types of FV can be analysed in relation to data about which types of FV the pupils received through the school FV programme.

### Implications for practice

The Boost project will provide new knowledge on the effectiveness of school-, family-and community-oriented strategies to increase FV intake among teenagers. This awareness may constitute a valuable basis for evidence-and practice-based recommendations to stakeholders. A strong environmental component like free provision of FV is highly dependent on funding and community priorities nationally and locally. An important part of the evaluation will be to sum up the costs of the various intervention components to inform stakeholders and health plan administrators. The analysis of the effect of the intervention in different social groupings will provide valuable information to politicians on possibilities to decrease social inequality in FV intake. To promote the implementation of the intervention at schools the curricular activities were designed to be implemented in different subjects so the teachers could share the assignment instead of only one teacher being responsible for the curriculum. The process evaluation will illuminate whether this is an approach to be recommended in health promotion initiatives.

It is challenging to reach schoolchildren in their leisure time due the large variety of activities and settings which they utilise. One fifth of the teenagers in the Boost study does not attend a youth-or sports club and sports-and youth clubs have very different facilities, policies and conditions. The process-and effect evaluation of the Boost leisure time intervention component will illicit valuable information about the opportunities and barriers for health promotion in this setting and may serve as a pilot study for more intensive, future interventions.

## Competing interests

The free FV school scheme was co-financed by 1) FDB (a Danish membership organisation which owns Coop, a Danish chain of grocery shops) and 2) the EU School Fruit Scheme through the Danish Food Industry Agency, the Danish Ministry of Food, Agriculture and Fisheries. These sponsors were not involved in the study design, data analysis or interpretation of data.

## Authors' contributions

RK is the principal investigator of the Boost study. RK conceived of and coordinated the study, contributed to its design, acquisition of data, statistical analysis, interpretation of data, and drafted the manuscript. AHC participated in collection of data, data cleaning, and statistical analyses and revised the manuscript critically. TSJ participated in the design of the study and collection of data, and revised the manuscript critically. AKA participated in the design of the study and collection of data, and revised the manuscript critically. AMC participated in the design of the study and revised the manuscript critically. PD conceived of the study, participated in its design, supervised the project group and revised the manuscript critically. All authors approve of the final version of the manuscript.

## Funding sources

The Boost study is part of Centre for Intervention Research in Health Promotion and Disease Prevention, National Institute of Public Health, University of Southern Denmark, Øster Farimagsgade 5A, 1353 Copenhagen K, Denmark. The Centre is funded by TrygFonden and the Danish Cancer Society. The Boost study is funded by a 5-year donation from TrygFonden including funding of each author and coverage of expenses related to intervention, implementation and evaluation. PhD scholarships for AKA and TSJ are co-financed by University of Southern Denmark. The free FV school scheme was co-financed by 1) FDB and 2) the EU School Fruit Scheme through the Danish Food Industry Agency at the Danish Ministry of Food, Agriculture and Fisheries. Copenhagen Food House contributed financially to the development of the teaching material.

## Pre-publication history

The pre-publication history for this paper can be accessed here:

http://www.biomedcentral.com/1471-2458/12/191/prepub
